# Restorativeness mediates the effect of a brief virtual reality mindfulness exposure with a multi-ethnic group in a natural environment on global identity salience: a pilot study with adolescents

**DOI:** 10.3389/fpsyg.2025.1593273

**Published:** 2025-06-26

**Authors:** Claudia Russo, Luciano Romano, Davide Clemente, Alessia Congiu, Roberta Rodelli, Claudia Navarini, Angelo Panno

**Affiliations:** ^1^Experimental and Applied Psychology Laboratory, Department of Health and Life Sciences, Università Europea di Roma, Rome, Italy; ^2^Geographic Research and Application Laboratory, Department of Human Sciences, Università Europea di Roma, Rome, Italy

**Keywords:** virtual reality, virtual natural environment, mindfulness, restorativeness, global identity, adolescence

## Abstract

**Introduction:**

Adolescents are increasingly exposed to global challenges, making it important to promote a sense of global identity—defined as a psychological connection with humanity as a whole. While scholars have highlighted the role of mindfulness and restorativeness in promoting global identity *per se*, there is a lack of studies hypothesizing their involvement in a unique framework and adopting virtual reality (VR) as a tool. This study aimed to verify, in a sample of adolescents, the indirect effect of the exposure to a VR video of a group of multi-ethnic youths practicing a brief mindfulness session – compared to a VR video of a group of multi-ethnic youths involved in a volleyball play – on global identity through restorativeness.

**Methods:**

A quasi-experimental between-subjects design was adopted, involving 94 Italian adolescents randomly assigned to one of two conditions: (i) a 360-degree VR video showing a group of multi-ethnic youths practicing mindfulness in a natural environment or (ii) a 360-degree VR video showing a group of multi-ethnic youths playing volleyball in the same environment. We hypothesized that the VR exposure to the group of multi-ethnic youths practicing a brief mindfulness session – compared to the VR exposure to the group of multi-ethnic youths involved in a volleyball play - could enhance restorativeness, which in turn is expected to increase global identity salience.

**Results:**

The results of the mediation analysis supported our hypothesis, showing that participants exposed to the mindfulness condition – compared to the participants exposed to the volleyball play condition - reported significantly higher restorativeness, which in turn led to an increase in global identity salience [point estimate = 0.12, SE = 0.08, 95% CI = (0.004, 0.317)].

**Discussion:**

Findings shed light on the underlying processes involved in the exposure to a brief mindfulness session within a multi-ethnic group played in a virtual natural environment on global identity salience, emphasizing the mediating role of restorativeness. These results provide significant insights into the self-concept construction, which is critical in adolescence. Practical implications and future research directions are discussed.

## 1 Introduction

In an increasingly globalized and multicultural world, it is essential to foster prosocial attitudes and increase the salience of global identity (Wang et al., 2023), conceptualized as the identification as a world citizen (Renger and Reese, 2017). Relying on the Social Identity Theory (SIT; Turner et al., 1987; Tajfel and Turner, 2010), a person’s self-concept consists of a set of cognitive representations at different levels of abstraction, ranging from self-identification as a unique and single individual to a member of global humanity (Wang et al., 2023). Adolescence is a critical life-span period during which significant developmental changes in the self-concept occur (Russo et al., 2019; Sebastian et al., 2008). As such, it might be considered an elite period to activate and increase the importance of global identity. The salience of global identity makes people more likely to act in favor of all human beings rather than only for other subgroups members based on nationality, ethnicity, religion, or race (Stürmer et al., 2016; Faulkner, 2018). Moreover, encouraging this kind of identification can foster different prosocial behaviors, ranging from willingness to volunteer or helping others in need (Reysen and Katzarska-Miller, 2013).

Recent evidence has highlighted the effectiveness of virtual reality (VR) as a tool to promote intergroup contact and foster prosocial behaviors also toward outgroup members (D’Errico et al., 2020; Tassinari et al., 2022; Yousefi et al., 2024). VR is an advanced technology that enables viewers to immerse themselves in virtual scenarios (Yu et al., 2018). VR has raised considerable interest among scholars as a potentially valuable tool for improving various aspects of human beings. Indeed, considering its features, VR has a wide range of possible uses, including, for example, entertainment, healthcare, and education (Yates et al., 2016; Joda et al., 2019; Rojas-Sánchez et al., 2022). Beyond its practical uses, VR can also serve as a tool for encouraging prosocial dimensions. Indeed, given its immersive nature, contents reproduced through VR have the potential to promote social good (Yousefi et al., 2024). Scholars have demonstrated the synergistic effects of virtual natural environment exposure combined with mindfulness in promoting positive outcomes through enhanced perceived restorativeness—defined as an individual’s subjective appraisal of an environment’s capacity to facilitate psychological restoration (Romano et al., 2024; Pasini et al., 2014). However, despite growing interest in this field, there is a lack of studies that have examined the potential of VR exposure to natural environments featuring a group of multi-ethnic youths engaged in mindfulness practice to enhance positive intergroup outcomes, such as global identity salience, via restorativeness mechanisms. To the best of our knowledge, existing literature has focused more on two distinct research trajectories than examining explicit mindfulness practices within virtual scenarios. In detail, studies examining mindfulness meditation conducted *in situ* within natural settings and its impact on environmental restorativeness and connectedness (e.g., Van Gordon et al., 2018), and research investigating the inherent mindfulness-promoting qualities of virtual natural environments and their capacity to foster both restorative experiences (e.g., Costa et al., 2019) and prosocial behaviors (Glennon and Barton, 2018). The present study addresses this gap by integrating these disparate research streams within a unified framework. Specifically, this study examines the underlying processes of the exposure to a group of multi-ethnic youths practicing mindfulness in virtual natural environments on global identity, with a particular focus on the mediating role of restorativeness.

### 1.1 Mindfulness and global identity

Mindfulness is defined as a mental state characterized by heightened awareness and focus on the present moment without any form of self-judgment (Kabat-Zinn, 2015). Originating from Buddhist practice, mindfulness has become widely popular in Western countries, and numerous studies have demonstrated its effectiveness in promoting psychological and physical well-being (e.g., Creswell, 2017; Goldberg et al., 2022; Goyal et al., 2014). Previous studies have further shown the role of mindfulness in fostering positive intergroup dimensions, such as readiness for intergroup contact and positive behavioral intention (Panno et al., 2018; Lillis and Hayes, 2007; Berger et al., 2018), leading to less implicit bias towards outgroup members (see Oyler et al., 2022 for a review). Recent studies have highlighted that mindfulness can lead to greater global identity, as connectedness with all of humanity is considered one of the constituent components of mindfulness practices (e.g., mind–body practices; Loy and Reese, 2019). Mindfulness sessions also seem to be effective when promoted via brief audio tracks through VR (Yildirim and O’Grady, 2020). Indeed, an immersive technology like VR can represent a beneficial resource for two main reasons. Firstly, the exposure to a multi-ethnic group of meditation represented through VR may give individuals access to peculiar groups that would not be easily accessible due to geographical distances, thus promoting the social component of mindfulness. Additionally, virtual environments can provide an accessible and engaging setting, making mindfulness more widely available (Costa et al., 2019).

### 1.2 The mediating role of restorativeness

While mindfulness itself has inherently restorative effects (Costa et al., 2019), these benefits appear to be amplified when practiced in natural settings—both real or virtual (Nisbet et al., 2019; Costa et al., 2020; see Spano et al., 2023 for a review). Restorativeness involves thoughts and perceptions of fascination, absorption, and being away (Berto, 2014). The concept of restorativeness is primarily explained by the Attention Restoration Theory (ART; Kaplan, 1995) and the Stress Reduction Theory (SRT; Ulrich et al., 1991). According to ART, natural environments—compared to urban settings—help alleviate mental fatigue by engaging involuntary attention. On the other hand, SRT suggests that natural settings are more effective than urban ones in facilitating psychophysiological recovery from stress (Ulrich et al., 1991; Kaplan, 1995; Berto, 2014). Indeed, urban environments are characterized by high levels of stimulation and stressors; on the contrary, nature promotes a replenishment from stressful states and a recovery from exhaustion resulting from the daily rhythms (Karmanov and Hamel, 2008; Menardo et al., 2021). Consistently, several studies have shown that virtual natural environments tend to elicit greater restorativeness compared to virtual urban settings (Schutte et al., 2017; Tabrizian et al., 2018; Mattila et al., 2020; Yu et al., 2020; see Spano et al., 2023 for a review). Moreover, recent experimental findings highlighted that virtual natural environments lead to positive psychological outcomes through restorativeness (Theodorou et al., 2023a; Romano et al., 2024). In addition, scholars have recently highlighted that even exposure to short mindfulness tracks *in vivo* or *in virtuo* natural environment can enhance nature’s benefits (Nisbet et al., 2019; Romano et al., 2024). A mindfulness-guided experience within a virtual natural setting may facilitate attentional focus on specific aspects of the environment and natural stimuli, thereby supporting the restorative process. Indeed, a greater awareness of the experience could amplify the perceived benefits of engaging in a restorative environment (Panno et al., 2020).

The relationship between restorativeness and global identity, on the other hand, can be better understood through the lens of three different theoretical frameworks, namely the Self-Expansion Model (Aron and Aron, 1997), the Identification with All Humanity framework (McFarland et al., 2012), and the Biophilia Hypothesis (Wilson, 1984). The Self-Expansion Model posits that individuals are motivated to enhance their efficacy and potential by including others—and, by extension, environments—within the self-concept. Natural and restorative environments, by offering opportunities for reflection, reduced self-focus, and cognitive replenishment, may serve as catalysts for such expansion. These environments not only allow for recovery from attentional fatigue and stress but also foster a sense of connectedness that transcends egocentric concerns. In other words, the restorative experience may facilitate the broadening of one’s self-boundaries to include a more inclusive sense of belonging and identification with all of humankind. This conceptual broadening aligns with the key aspects of the Identification with All Humanity framework, which emphasizes the cognitive and affective processes by which individuals identify themselves as part of a superordinate ingroup. Complementing this perspective, the Biophilia Hypothesis posits that humans are biologically predisposed to form bonds with all living beings—a concept that might favor global identity salience. This process appears to be particularly facilitated by the so-called ‘focus upon life’, an aspect that Barbiero and Berto (2018) have compared to fascination, a core characteristic of restorativeness. Specifically, according to the authors, the capacity to attend to the restorative qualities of the environment represents a learned yet biologically determined mechanism that promotes connection and bonding with living beings. In this vein, previous evidence suggests that the benefits related to nature’s exposure positively affect prosocial outcomes, such as increased feelings of inter-connectedness (Neill et al., 2019) and social cohesiveness (Holtan et al., 2015) (see Goldy and Piff, 2020 for a review).

Taken together, the Self-Expansion Model, the Identification with All Humanity framework, and the Biophilia Hypothesis (Aron and Aron, 1997; McFarland et al., 2012; Wilson, 1984) offer a complementary perspective on how restorative experiences in nature can favor a broad, inclusive identity. Specifically, these frameworks suggest that such experiences can reduce self-focus, foster connection with others and with all living beings, and enhance a sense of unity with humanity. These processes are particularly relevant during adolescence, a stage marked by identity exploration and the search for meaningful social bonds (Branje et al., 2021; Crocetti, 2017). In this context, restorativeness may foster the development of global identity by encouraging a deeper sense of human interconnectedness.

### 1.3 The present study

Drawing on previous research that showed a positive effect of mindfulness on both inter-group relations and environmental psychology outcomes (e.g., Panno et al., 2018; Ray et al., 2021; Rehman et al., 2023), the main aim of this study was to examine the indirect effect of exposure to two natural virtual reality scenarios involving the same multi-ethnic group of youths (while experiencing a mindfulness audio-track vs. without experiencing a mindfulness audio-track) on global identity through restorativeness. Specifically, the two virtual scenarios consisted of (i) a 360-degree video played in virtual reality including a group of multi-ethnic youths experiencing a mindfulness audio-track in a natural environment; (ii) a 360-degree video played in virtual reality including a group of multi-ethnic youths playing volleyball in a natural environment.

We hypothesized that the virtual reality exposure to the 360-degree video including a group of multi-ethnic youths experiencing mindfulness audio-track in a natural environment – compared to the virtual reality exposure to the 360-degree video including a group of multi-ethnic youths playing volleyball in the same natural environment - would enhance participants’ restorativeness and this, in turn, will lead to an increase of global identity salience.

## 2 Materials and methods

### 2.1 Participants

The current study is based on a quasi-experimental between-subject design recruiting a sample of Italian adolescents. A total of 7 classes from a high school located in Lazio, Italy, were involved in the study. Based on a previous study (Theodorou et al., 2023b), we estimated at least 30 participants for each of the two conditions before collecting our data. Therefore, an initial sample of 109 high school students (women = 60.6%) aged 14 to 16 (*M* = 14.55; SD = 0.53) was collected.

However, of these 109 high school students, a total of 9 participants declared ethnic origins other than Italian and, for this reason, were excluded from the final sample. Finally, the other 6 participants were excluded from the final sample after assessing for the attentional check (i.e., “In the video you saw, the group of people was”: “A multi-ethnic group of people” or “An Italian only group of people”).

Therefore, the final sample comprised 94 Italian high school students aged 14 to 16 (*M* = 14.53; SD = 0.50). Of these, 62.8% identified themselves as girls and 37.2% as boys. All adolescents were born in Italy, had Italian parents, and had Italian citizenship. A total of 40 students were assigned to the first condition (i.e., a 360-degree video including a group of multi-ethnic youths playing volleyball in a natural environment) (Mage = 14.27; SD = 0.45; *F* = 45%), and a total of 54 students were assigned to the second condition (i.e., a 360-degree video including a group of multi-ethnic youths practicing a brief mindfulness session in a natural environment) (Mage = 14.73; SD = 0.44; *F* = 75.9%). Further details on the two experimental conditions are given below.

### 2.2 Experimental conditions

To test our hypothesis, participants were assigned to two different experimental conditions, which consisted of exposure to two different virtual reality scenarios through head-mounted displays (HMDs) for virtual reality, namely Oculus Quest 2. In detail, the two scenarios were 360-degree videos made by the researchers with a 360-degree shooting camera (Insta360 X2). Both the virtual scenarios were registered in a green area located in Villa Pamphili, a public green park in Rome, Italy (coordinate: 41.887657, 12.453529) on two different days with similar climate conditions (i.e., sunny) during the morning hours. Specifically, one 360-degree video (i.e., assigned to the control group) represented a group of multi-ethnic youths (i.e., an Italian girl, a Chinese girl, a Peruvian boy, and an African boy) sitting close together on the grass to form a semicircle and playing volleyball (i.e., participants quickly exchanged a ball with each other). The second 360-degree video (i.e., assigned to the experimental group) consisted of a group of multi-ethnic youths (i.e., the same Chinese girl, Peruvian boy, and African boy of the first video and an Italian girl with physical characteristics similar to the girl in the first video) practicing a short mindfulness session. Once finalized, the 360-degree videos were implemented in two head-mounted displays (HMDs) for virtual reality, namely Oculus Quest 2. The mindfulness audio-track adopted was developed in line with previous research (e.g., Segal et al., 2002; McHugh and Wood, 2013; Eisenbeck et al., 2018) and is based on breath-centered mindfulness meditation. The outline includes the following instructions: (a) adopt a comfortable sitting position; (b) direct attention to bodily sensations; (c) focus on the breath and accompanying sensations; (d) bring attention back to the breath if the mind wanders; and (e) practice self-compassion and gratitude for the present moment. Based on Menhart and Cummings' (2022) study, which found that participants rated mindfulness meditation as more beneficial when delivered by a female voice, we chose to have the track recorded by a professional voice actress.

Noteworthy, the volleyball play condition was selected as an *active control* for several reasons. First, it matched the experimental condition in terms of setting (i.e., the same green area), number and composition of characters (i.e., multi-ethnic group of peers), and duration, thus controlling for visual complexity, environmental exposure, and intergroup representation. Second, volleyball was chosen as a familiar, socially engaging activity that is plausible in an outdoor setting (e.g., Weng and Chiang, 2014) and has been previously used in VR paradigms examining social inclusion (e.g., Williams and Jarvis, 2006). More relevant, the control condition was designed to isolate the unique contribution of the mindfulness practice in the experimental condition, while maintaining comparable levels of exposure to nature and multi-ethnic components. In more detail, both conditions involve elements leading to restorativeness (i.e., the green area) or prosocial interpretation (i.e., exposure to a multi-ethnic group), but only the experimental condition explicitly includes mindfulness instructions to be compared to the volleyball play condition. Therefore, we hypothesized that the mindfulness practice would be the key mechanism affecting the psychological outcomes of interest in the current study. This approach aligns with best practices in experimental design, where structurally equivalent active control conditions are used in randomized between-subjects designs to isolate the specific contribution of key mechanisms (e.g., Britton et al., 2014; Hughes et al., 2023; MacCoon et al., 2012, 2014).

### 2.3 Procedure

The research protocol follows the Declaration of Helsinki of 1964 and its latest versions, and the method applied in the study was approved by the Institutional Ethics Committee of the Università Europea di Roma, Rome, Italy (prot. no.02/2024). The research protocol received the approval of the school council as well as of the school principal of the high school involved in the study. The research was conducted from October to April 2024, and the quasi-experimental procedure was set as follows. In the high school that joined the research, a classroom was set up and used solely for the purpose of the study. Two students at a time were called to take part in the experiment. For the exposure to the experimental conditions, two mats were placed at the two extreme sides of the classroom. Before the experiment began, floor boundaries were placed around the mats to ensure that they remained in the same position throughout the experiment. Moreover, two desks with two laptops were placed at either end of the classroom so that students could fill out the post-exposure questionnaire. Eligible participants were accompanied to the designated room by a member of the research team. Once in the room, students were invited to sit on the mat and helped to wear the Oculus Quest 2, already set up by research assistants for 360-degree video viewing. The participants were then exposed to one of the two experimental conditions: (a) the VR 360-degree video including a group of multi-ethnic youths playing volleyball in a natural environment; (b) the VR 360-degree video including a group of multi-ethnic youths experiencing a brief mindfulness audio-track in a natural environment. Based on previous evidence (e.g., Romano et al., 2024), the duration of the exposure to virtual conditions was set at around 4 min. During the VR exposure, research assistants were present in case they were needed. After this phase, the participants were asked to complete the post-exposure questionnaire. Anonymity and confidentiality standards were ensured for all participants at every data collection stage.

### 2.4 Measures

After the exposure to the VR scenarios, a post-exposure questionnaire was administered, including our dependent variables (i.e., restorativeness and global identity), state mindfulness, and socio-demographic information (i.e., age, gender, nationality, place of birth, and parental place of birth).

Restorativeness. Consistent with a previous study (Theodorou et al., 2023a), we used 7 items from the short version of the Perceived Restorativeness Scale (PRS-11) in its Italian version (Pasini et al., 2009, 2014). An example of an item is “The place shown is fascinating.” The response scale ranged from 1 (not at all) to 5 (very much). The total score was used (*α* = 0.73; *ω* = 0.75).

Global identity. Based on previous studies (Renger and Reese, 2017), we used a single item to assess the post-exposure global identity. Specifically, students were asked to indicate their degree of agreement by thinking about the lived experience, i.e., viewing the video in VR. The item was “I saw myself as a global citizen.” The response scale ranged from 1 (not at all) to 5 (very much).

State mindfulness. The State Mindfulness Scale (SMS; Tanay and Bernstein, 2013) was used to assess state mindfulness post-exposure. The items were translated into Italian using a back-translation method. The scale is composed of 21 items: 15 items for the State Mindfulness Mind dimension (e.g., “*I noticed emotions come and go*”) and 6 items for the State Mindfulness Body dimension (e.g., “*I felt in contact with my body*”). We modified the scale instructions, asking participants to refer to the present moment they were experiencing. The Likert scale ranged from 1 (not at all) to 5 (very much). We computed the total score (α = 0.95; ω = 0.95).

## 3 Results

### 3.1 Preliminary analysis

We conducted a one-way ANOVA to examine whether the two groups showed differences in state mindfulness after exposure to the experimental conditions. The results revealed significant differences between groups [*F* (1,92) = 9.008, *p* = 0.003]. In detail, participants who were assigned to the mindfulness condition reported higher levels of state mindfulness (*M* = 3.81, SD = 0.68) compared to the participants assigned to the condition without the mindfulness session (*M* = 3.35, SD = 0.80).

### 3.2 Mediation analysis

**Figure 1:**
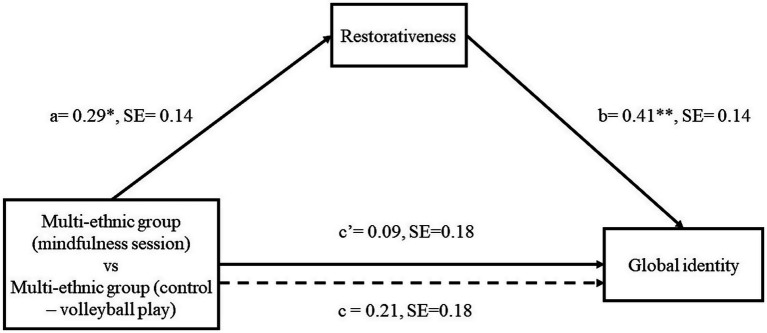
Graphical representation of the meditation model results. *p < 0.05, **p < 0.01. The dotted line denotes the effect of experimental conditions on global identification when restorativeness is not included as a mediator; a, b, c, and c’ are unstandardized regression coefficients. 1 = Multi-ethnic group (control – volleyball play), 2 = Multi-ethnic group (mindfulness audio track).

The results of the mediation model are reported in Figure 1.

In the first step, the dummy coded variable (i.e., 1 = the group of multi-ethnic youths playing volleyball in a natural environment, 2 = the group of multi-ethnic youths experiencing mindfulness in a natural environment) was entered as predictor of restorativeness. The results of this model explained 5% of the variance of restorativeness (*R*^2^ = 0.05, *F* = 4.31, *p* = 0.04). Specifically, the effect of the virtual reality experimental condition including a group of multi-ethnic youths experiencing a brief mindfulness audio-track in a natural environment – compared to the virtual reality experimental condition including a group of multi-ethnic youths playing volleyball in a natural environment - on restorativeness was positive and significant (see Figure 1, path a); thus, showing that it was more effective in enhancing restorativeness. In the second step, both dummy coded variable and restorativeness were included as predictors, while global identity was inserted as the outcome. This model explained 10% of the variance of global identity (*R*^2^ = 0.10, *F* = 4.75, *p* = 0.01). The indirect effect of the virtual reality experimental condition including a group of multi-ethnic youths experiencing a brief mindfulness audio-track in a natural environment, on global identity through restorativeness was positive and significant [point estimate = 0.12, BootSE = 0.08, (95% percentile BootCI = 0.004, 0.317)], compared to the virtual reality experimental condition including a group of multi-ethnic youths playing volleyball in a natural environment.

In detail, as shown in Figure 1, results revealed that the exposure to the mindfulness condition – compared to the exposure to the volleyball condition - led to higher restorativeness (path a in Figure 1), which in turn led to higher global identity (see path b in Figure 1). Moreover, our results showed that both direct and total effects were not significant (see path c’ and c Figure 1). Nonetheless, according to Hayes (2009) and other scholars (e.g., MacKinnon et al., 2000), researchers do not need to establish a significant total effect before examining indirect effects. As highlighted by Hayes (2009, pp. 414–415), the lack of deepening indirect effects in the absence of a total effect may result in overlooking meaningful, relevant, or informative mechanisms through which X may influence Y. Taken together, these results revealed that the exposure to the virtual reality experimental condition with the mindfulness audio-track was more effective in enhancing global identity through restorativeness than the control condition.

Finally, as a further check and following an exploratory approach, the moderating role of gender (coded as 1 = Women and 2 = Men) in the hypothesized mediation model was verified. Specifically, the moderation of gender was tested on the direct path (i.e., experimental conditions – global identity). Results revealed that the interaction term conditions*gender on global identity was not significant [b = 0.37, SE = 0.39, 95%CI (−0.415, 1.174)]. However, the indirect effect of the exposure to the mindfulness condition – compared to the exposure to the volleyball condition on global identity through restorativeness remained significant [point estimate = 0.12, BootSE = 0.08, 95%BootCI (0.002, 0.322)].

## 4 Discussion and conclusions

The present study sought to investigate the indirect effect of virtual reality exposure to a natural virtual environment in which a multi-ethnic group of youths experienced a mindfulness session – compared to virtual reality exposure to a natural environment in which a multi-ethnic group of youths played volleyball – on global identity through restorativeness in adolescence.

Our findings confirmed our hypothesis, showing that the virtual natural scenario with the brief mindfulness session in a multi-ethnic group – compared with the other virtual natural control condition - was more effective in enhancing restorativeness, which in turn led to an increase in global identity. These results can be partially understood within the framework of the Attention Restoration Theory (ART; Kaplan, 1995) and Stress Reduction Theory (SRT; Ulrich et al., 1991). These theories point out that natural environments allow people to overcome mental fatigue, restoring cognitive resources on one side and reducing stress levels on the other (Jimenez et al., 2021). However, our findings suggest that the combination of natural environments and mindfulness enhances nature’s benefits (Nisbet et al., 2019), yielding higher levels of restorativeness. Mindfulness can improve the restorative qualities of nature (Costa et al., 2020) in different ways. Being exposed to a mindfulness audio track in a natural environment might lead people to perceive nature more intensely, increasing both the feeling of being connected with it and the perception of the regenerative beauty of nature (Van Gordon et al., 2018). Thus, consistent with a recent study (Romano et al., 2024), it seems that even brief sessions of mindfulness in virtual natural environments can improve the benefits associated with nature exposure.

Furthermore, considering our results, these benefits, in turn, increased the salience of global identity. This finding is in line with the Social Identity Approach (Turner et al., 1987; Tajfel and Turner, 2010), according to which the facets of the social context in which people act and interact have an influential role in determining which level of cognitive representations of the self will be activated. By its inherent characteristics, mindfulness enables people to focus their attention on the present moment, increasing the restorative properties of the natural environment (Costa et al., 2019; Nisbet et al., 2019) and generating a state of well-being and recovery from stress (Berto, 2014). As previously highlighted, there is a strong association between well-being and social identification (Steffens et al., 2017). Thus, this increment in the well-being levels allows, in turn, a greater identification as citizens of the world.

Although this study presents new insights and strengths, the exploratory nature of the study resulted in a number of limitations that need to be underlined. First, since we adopted a quasi-experimental design, we cannot point out causal relationships among these variables. Another relevant limitation is the absence of pre-intervention assessments for the main constructs under study, including state mindfulness. This limits the ability to determine whether the differences observed between groups after the intervention can be attributed to the experimental manipulation or were already present at baseline. Moreover, due to the quasi-experimental nature of the research and the constraints of conducting the study within the school context, participants were not randomly assigned to conditions, which further limits the internal validity of the findings. Despite these limitations, the significant differences observed in state mindfulness between the groups post-intervention provide preliminary evidence suggesting the potential effectiveness of the experimental condition. Future studies should employ randomized controlled designs and include baseline measurements to allow for more robust causal inferences and a clearer understanding of the experimental condition’s impact. Furthermore, our study assessed immediate post-exposure effects. Thus, future works should include multiple measurements at different times. Indeed, the use of repeated measurements can provide relevant information on whether and for how long the effect obtained through exposure to the experimental condition with mindfulness can last over time, reinforcing the results obtained. Another limitation concerns the lack of a formal pre-test to validate the equivalence between virtual reality (VR) recordings and real-life natural settings. Although the 360-degree videos were filmed in an actual natural environment—thus ensuring high visual and contextual fidelity—future research should include validation procedures to more robustly assess ecological equivalence. Nonetheless, prior studies have shown that immersive 360° VR experiences can elicit psychological responses comparable to *in vivo* exposure (e.g., Spano et al., 2023; Rosendahl and Wagner, 2024). Furthermore, participants were ethnically homogeneous (Italian), and the sample was gender-imbalanced, with a majority of female adolescents. This may limit the generalizability of the findings to more diverse populations. Cultural background and gender identity can shape both the perception of mindfulness experiences and the salience of global identity, particularly in adolescence, a developmental phase marked by active identity exploration (e.g., Crocetti, 2017). The present study intentionally focused on a homogeneous sample of Italian adolescents living in Italy, with the aim of investigating the effects of the VR mindfulness exposure within the majority group. While this approach allowed us to examine the exposure’s effects in a culturally and contextually specific group, it also limits the generalizability of our findings. Specifically, the ethnic homogeneity of the sample precludes any inference about how adolescents from minority or migrant backgrounds might respond to the same exposure. Future studies should explicitly examine the effects of this VR exposure in more ethnically diverse samples to explore potential cultural or contextual variations in effectiveness.

Moreover, while the indirect effect and explained variance of global identity (10%) are modest, this is not unexpected given the complexity of global identity. Small effects are common in psychological research, especially when targeting complex constructs such as global identity, which are likely influenced by a broad array of individual, contextual, and cultural factors. Therefore, considering our study design and the complexity of the phenomenon, we believe that even small effects should be discussed, as suggested by Funder and Ozer (2019).

In line with prior research in psychology, including environmental studies (e.g., Bergkvist, 2015; Cologna et al., 2021; Wanous et al., 1997; Whitmarsh et al., 2022), a single-item measure was adopted to assess global identity. While single-item measures may not capture the multidimensionality of complex constructs, they are often employed in youth research to reduce cognitive fatigue and enhance focus (Matthews et al., 2022). However, we acknowledge that this approach may limit the interpretive depth of the findings and future studies should consider replicating our findings by adopting multi-item scales (e.g., the Identification With All Humanity Scale; McFarland et al., 2012). Finally, it might be interesting in future studies to assess whether the findings can be achieved involving adults, who, contrary to adolescents, should have a more developed self-concept (Sebastian et al., 2008; Russo et al., 2021).

Despite these limitations, to the best of our knowledge, this is the first study that has deepened the underlying mechanisms of the effects of a virtual reality scenario with a group of multi-ethnic youths experiencing a brief mindfulness session in a natural environment on global identity through restorativeness. Furthermore, no previous studies have examined the abovementioned relationships in a sample of adolescents and using virtual reality before. Moreover, to our knowledge, the relationship between restorativeness and global identity was not explored in previous research. Increased salience of global identity can lead to prosocial attitudes and behaviors (Stürmer et al., 2016; Faulkner, 2018), suggesting that the combination of virtual natural environments and mindfulness can be beneficial in educational contexts such as schools. Since the role of schools is to shape future responsible and tolerant citizens, these findings pave the way for the possibility of introducing virtual reality mindfulness sessions in natural environments as part of school curricula. Furthermore, it might be interesting to develop and evaluate a virtual reality-based nature mindfulness intervention to strengthen - and not only activate - adolescents’ global identity.

## Data Availability

The raw data supporting the conclusions of this article will be made available by the authors, without undue reservation.
