# Correction: Restorativeness mediates the effect of a brief virtual reality mindfulness exposure with a multi-ethnic group in a natural environment on global identity salience: a pilot study with adolescents

**DOI:** 10.3389/fpsyg.2025.1662901

**Published:** 2025-07-30

**Authors:** Claudia Russo, Luciano Romano, Davide Clemente, Alessia Congiu, Roberta Rodelli, Claudia Navarini, Angelo Panno

**Affiliations:** ^1^Experimental and Applied Psychology Laboratory, Department of Health and Life Sciences, Università Europea di Roma, Rome, Italy; ^2^Geographic Research and Application Laboratory, Department of Human Sciences, Università Europea di Roma, Rome, Italy

**Keywords:** virtual reality, virtual natural environment, mindfulness, restorativeness, global identity, adolescence

In the acknowledgments, the following text was unintentionally omitted from the original article.

## Acknowledgments

We thank Ettore Majorana High School in Latina, the Dean Prof. Domenico Aversano, and Profs. Marina Santoro, Daniele Ribatti, Natalina Porcelli, Bernardo Mele, Enrica Teti, Immacolata Pezzella, Emanuela Trani, Claudia Pernarella, Valentina Pugliano, Daniela Pimpinella, all the teachers, Caterina Coughlin, Maria Arena, Eleonora Leonardi, and Emanuela Loreti (interns and graduands from the Experimental and Applied Psychology Laboratory) who supported the project.

The original version of this article has been updated.

